# Durable benefit and slowdown in tumor growth dynamics with erdafitinib in a FGFR3-TACC3 fusion-positive IDH-wild type glioblastoma

**DOI:** 10.1093/noajnl/vdae139

**Published:** 2024-08-06

**Authors:** Santiago Cabezas-Camarero, Rebeca Pérez-Alfayate, Carmen Polidura, María Natividad Gómez-Ruiz, Lidia Gil-Martínez, Isabel Casado-Fariñas, Jorge Bartolomé, Pedro Pérez-Segura

**Affiliations:** Medical Oncology Department, IOB Institute of Oncology-Madrid, Madrid, Spain; Medical Oncology Department, Instituto de Investigación Sanitaria San Carlos (IdISSC), Hospital Clínico Universitario San Carlos, Madrid, Spain; Neurosurgery Department, Hospital Clínico Universitario San Carlos, Madrid, Spain; Radiology Department, Hospital Clínico Universitario San Carlos, Madrid, Spain; Radiology Department, Hospital Clínico Universitario San Carlos, Madrid, Spain; Radiology Department, Hospital Clínico Universitario San Carlos, Madrid, Spain; Pathology Department, Hospital Clínico Universitario San Carlos, Madrid, Spain; Medical Oncology Department, Instituto de Investigación Sanitaria San Carlos (IdISSC), Hospital Clínico Universitario San Carlos, Madrid, Spain; Medical Oncology Department, IOB Institute of Oncology-Madrid, Madrid, Spain; Medical Oncology Department, Instituto de Investigación Sanitaria San Carlos (IdISSC), Hospital Clínico Universitario San Carlos, Madrid, Spain

**Keywords:** erdafitinib, FGFR3-TACC3 fusion, glioblastoma

## Abstract

FGFR3-TACC3 fusion-positive IDH-wild-type (IDH-WT) glioblastoma (GB) is a rare GB subtype occurring in approximately 3% of cases. It is clinical behavior and molecular profile is different from those of fusion-negative IDH-WT GBs. Evidence on the role of FGFR inhibitors in FGFR-altered gliomas is limited. We present the case of a patient with a FGFR3-TACC3 fusion-positive IDH-WT GB that at its second recurrence was treated with the FGFR inhibitor erdafitinib through a compassionate use program. Although no objective response was achieved, an overt deceleration in tumor growth was evidenced and the patient remained on treatment for 5.5 months.

 Glioblastoma (GB), isocitrate dehydrogenase (IDH) wild-type (WT) is the most common primary malignant brain tumor with a median overall survival (OS) of 15–20.9 months despite maximal safe surgical resection followed by radiotherapy (RT) and concurrent and adjuvant temozolomide (TMZ) with or without the addition of tumor treating fields.^1,2^ Fibroblast growth factor receptor (FGFR)-Transforming acidic coiled-coil-containing gene (TACC) fusion-positive IDH-WT GB has been informally recognized as a distinct entity in recent years occurring in 3%–3.5% of IDH-WT GBs.^3–6^ They lack specific morphological or radiological characteristics. Therefore molecular studies must be performed in order to identify FGFR-fusion positive IDH-WT GB. FGFR-TACC fusions in gliomas have been reported to be mutually exclusive with EGFR and IDH alterations, commonly cooccur with CDK4 and MDM2 amplifications, and have been associated with a better prognosis compared to non-FGFR-altered IDH-WT GBs.^3,4^ Erdafitinib is a small-molecule, pan-FGFR inhibitor targeting FGFR 1–4 alterations, that gained full FDA approval in January 2024 for second-line treatment of FGFR3-altered advanced urothelial cancer who had progressed to at least one prior systemic therapy, after demonstrating a statistically significant improvement in OS, PFS and ORR in the study BLC3001 Cohort 1.^7^ The phase III THOR trial demonstrated a significant gain in OS for patients treated with erdafitinib compared to chemotherapy after progression to anti-PD(L)1 agents (OS: 12.1 vs 7.8 months; HR 0.64; 95% CI: 0.47–0.88, *P* = .005).^8^ However, the evidence on the efficacy and safety of erdafitinib and other FGFR inhibitors in CNS tumors is currently very limited.^4,5,9–11^

## Methods

### Ethical Considerations

The investigators obtained informed consent from the patient to publish information and clinical images. This study was approved by the Institutional Review Board of Hospital Clínico Universitario San Carlos (IRB code 16/549-E).

### Immunohistochemistry

IDH1-R132H antibody (clone H09; 1:20, IDAH09, Dianova, Hamburg), GFAP (clone 6F2; 1:100), TP53 (clone DO-7; 1:50), and Ki67 (clone MIB-1; 1:100) antibodies (Dako North America, Carpintería, CA) were used for the expression of GFAP, IDH1-R132H, TP53 and Ki67 in an autostainer Dako Omnis (Agilent Technologies, Inc.), as previously described.^12^

### Next Generation Sequencing

A Next Generation Sequencing (NGS) multigene panel (FoundationOne CDx, Roche Diagnostics, Inc.) was used for molecular analysis of the tumor biopsies collected at initial diagnosis in September 2019, and during the first and second rescue surgeries from April 2022 and June 2023, respectively.^13^

### Radiological Tumor Assessment

Tumor evaluation was performed with MRI following the response assessment in neuro-oncology criteria.^14^ Tumor lesion volumes were calculated using FSL FMRI (Analysis Group, FMRIB, Oxford, UK), after manual segmentation of the enhancing tumor lesions on 3D postgadolinium T1-weighted images.

## Case Report

**Figure 1.**
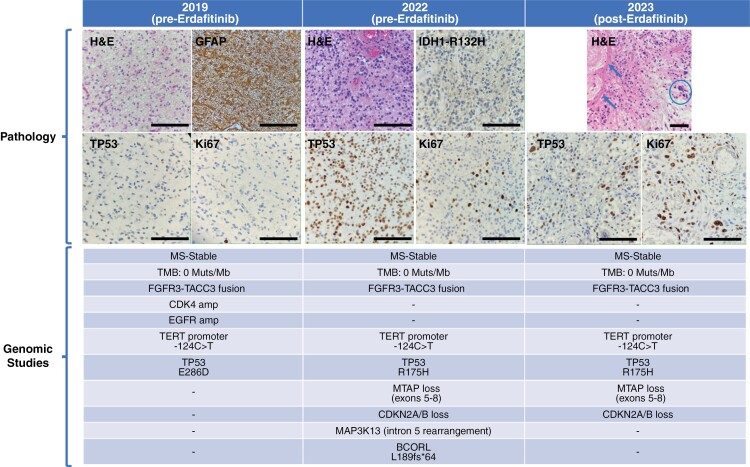
Pathology characteristics and results of genomic studies in each of the tumor biopsies performed. Pathology findings: The 2019 tumor biopsy shows a low-grade glioma, expressing GFAP and with near-negative TP53 expression and a very low proliferative index (Ki67 = 1%–2%). Scale bar 100 mm (200× magnification). The 2022 tumor biopsy shows a low-grade glioma IDH1-R132H negative, diffusely TP53-mutant and with a higher Ki67. Scale bar 100 mm (200× magnification). The 2023 tumor biopsy shows a high-grade histology with necrosis and cellular pleomorphism and a higher Ki67 than in the prior biopsies. Scale bar 100 mm (H&E: 100× magnification; TP53 and Ki67: 200× magnification). Genomic studies: The 3 tumor biopsies demonstrate a microsatellite stable (MSS) tumor, with a low TMB (0 muts/Mb), harboring a FGFR3-TACC3 fusion, a TERT promoter mutation (−124C>). The 2022 and 2023 biopsies show the same TP53 mutation (R175H), CDKN2A/B loss and MTAP loss (exons 5–6). GFAP: gliofibrillary acid protein, H&E: hematoxylin and eosin, IDH1-R132H: IDH1-R132H mutation, Ki67 proliferative index, TP53: TP53 mutation. Arrows and circle indicate areas of necrosis and cellular pleomorphism, respectively.

**Figure 2.**
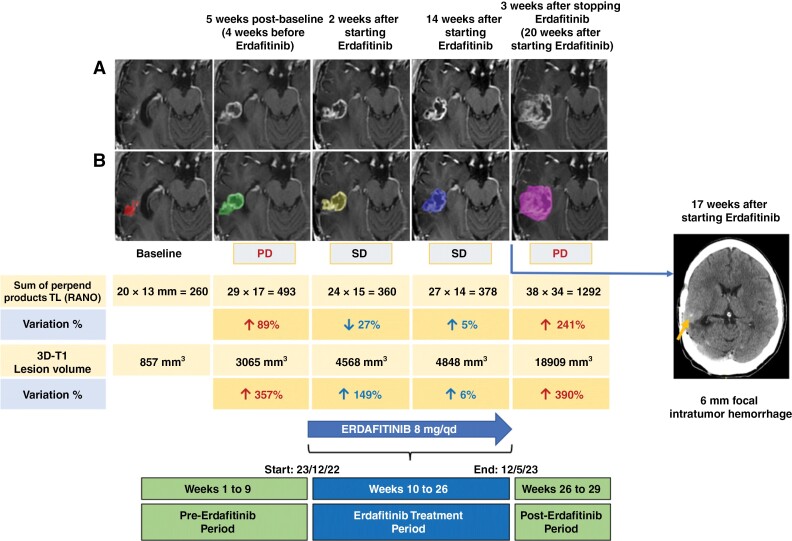
Tumor growth dynamics before, during and after treatment with erdafitinib. Immediately before starting erdafitinib an 89% tumor growth according to Response Assessment in Neuro-Oncology (RANO) criteria was seen between 2 MRIs taken 5 weeks apart in October and November 2022. However, in the next MRI reevaluation, 2 weeks after erdafitinib had been started, a 27% reduction in bidimensional tumor size is seen. In the next MRI performed 4 months after the start of erdafitinib a 5% increase in tumor size can be seen. Nevertheless, in early June 2023, 3 weeks after stopping erdafitinib a brisk increase in tumor size is evident (A). The 2-dimensional T1 (3D-T1) tumor volume was also measured showing a slowdown in tumor growth during treatment with erdafitinib compared to the pre- and post-erdafitinib MRIs (B). The upper row shows the T1 postgadolinium images of the tumor lesion in the right parietal lobe for bi-dimensional size measurement according to RANO criteria (A). The lower row shows the same T1 postgadolinium axial images with superimposed colors over the tumor area for volume calculation. Each color represents a different time point (B). Arrow points to the 6 mm hemorrhagic focus on the CT scan from May 2023. MRI, magnetic resonance imaging; PD, progressive disease; SD, stable disease.

In September 2019, a 38-year-old male, carrier of the germline prothrombin mutation G20210A and harboring a lupic anticoagulant, with no other relevant medical history, underwent a partial tumor resection of a 2 cm right parietal non-enhancing lesion. Pathology informed of an IDH-WT GB (Figure 1). However, histomorphology was reminiscent of low-grade glioma (LGG) with a moderate increment in GFAP+ astrocytic cellularity, scant atypia, and lack of mitotic figures, microvascular proliferation, or necrosis. Ki67 proliferation index was 1%–2%. The patient received standard radiotherapy with concurrent (75 mg/m2/qd) and adjuvant TMZ (150–200 mg/m2 days 1–5/q4wk for 6 cycles), which he finished in June 2020. In March 2022, a tumor relapse was diagnosed, and the patient underwent a complete tumor resection. Pathology informed of an IDH-WT GB, with some histological characteristics indicative of transformation to a more aggressive disease: Cellular pleomorphism and atypia, one atypical mitotic figure, and a small focus of atypical microvascular proliferation, but without necrosis. Ki67 proliferation index was 5%–10%. NGS of the tumor biopsies from the initial diagnosis and from the relapse from March 2022 revealed that the tumor was IDH1 and IDH2 wild type and harbored an FGFR3-TACC3 fusion (Figure 1, Supplementary Table S1). In October 2022, a new tumor relapse was evidenced. Being neurologically intact with no apparent deficits, close follow-up ensued. However, tumor growth accelerated with progressive disease occurring in November 2022. Therefore, in December 2022, treatment with the FGFR-inhibitor erdafitinib through a compassionate use program (Johnson & Johnson Innovative Medicine), was started at 8 mg/qd that he tolerated well except for grade 1 hyperphosphatemia and diarrhea, and grade 2 onycholysis, paronychia, and onychodystrophy. MRI in January and April 2023 showed stable disease. However, in May 2023 the patient was admitted to the ER after experiencing mild left hand and forearm apraxia that rapidly resolved with 10 mg of intravenous dexamethasone. An urgent CT scan showed a 6 mm intratumor area concordant with an acute hemorrhagic focus. Due to the potential relation with the tumor bleeding, treatment with erdafitinib was stopped. A brain MRI performed three weeks after stopping erdafitinib showed an overt tumor progression. A clear slowdown in tumor growth dynamics was evidenced during treatment with erdafitinib compared to the periods immediately prior to the start and following the stop of erdafitinib (Figure 2, Supplementary Figure S1).

In June 2023, once erdafitinib was abandoned, the patient underwent surgery with partial tumor resection followed by reirradiation (40 Gy in 15 fractions) with concurrent and adjuvant TMZ, finishing the sixth adjuvant cycle in February 2024. Pathology informed of an IDH-WT GB, with typical high-grade histomorphological features. There was widespread necrosis, microcalcifications, atypical microvascular proliferation, marked cellular pleomorphism, and inflammation by polymorphonuclear cells. Ki67 proliferation index reached 12%. NGS of this third tumor biopsy revealed the tumor continued to be IDH-wild type and still harbored the FGFR3-TACC3 fusion described in the two previous biopsies (Figure 1, Supplementary Table S1). In early March 2024, mild hemiparesis appeared with a brain MRI showing progressive disease. A new line with fotemustine 75 mg/m2/q2wk was started without response and the patient died due to tumor progression in April 2024, 57 months after initial diagnosis.

## Discussion

In the phase II basket RAGNAR study, among 30 patients with high-grade glioma (HGG), 97% harbored FGFR fusions and only 3% FGFR mutations. Among 7 patients with LGG, 43% harbored FGFR fusions and 57% had FGFR mutations. In HGG, the most frequent FGFR alterations occurred in FGFR3 (27/30: 90%) while in LGG alterations in FGFR1 (5/7: 71%) were most common. ORR and disease control rate (DCR) for the whole glioma cohort was 13.5% and 59.5%, respectively. ORR and DCR were higher among LGG (29% and 71%, respectively) compared to patients with HGG (10% and 57%, respectively).^9^ Interestingly, in the RAGNAR study, a 26-year-old female with a FGFR1-K656E fusion-positive LGG, presented a complete tumor response lasting for at least 21.7 months^9^ (Table 1).

**Table 1.** Summary of Most Relevant Studies With FGFR Inhibitors in Patients With FGFR-Altered Gliomas

Author (year)	Study type	*N*	Agent	ORR	PFS	OS
Di Stefano (2015)^4^	Case report	2	Erdafitinib	SD	115 and 134 days	Both pts alive at last follow-up
Wang (2021)^11^	Case report	1	Anlotinib + TMZ	PR	17 m	Alive at last follow-up
Sait (2021)^15^	Single center study	5	Debio1347	DCR 80%	—	—
McDonald (2022)^5^	Case report	1	Erdafitinib	No response	—	—
Lassman (2022)^16^	Phase II	26	Infigratinib	3.8%	1.7 m (6-m PFS 16%)	6.7 m
Pant (2023)^9^	Phase II	37	Erdafitinib	ORR/DCR HGG: 10%/57% LGG: 29%/71% All gliomas: 13.5%/59.5%	—	—
Padovan (2023)^10^	Retrospective study	4	Erdafitinib	2 SD 2 PD DCR: 50%	2.2 m	—
Spanggaard (2023)^17^	Phase II	13	Pemigatinib	DCR: 46.2%	—	6.1 m
Gong (2024)^18^	Phase II	2	Erdafitinib	2 SD	—	—
Liu (2024) ^19^	Case report	1	Pemigatinib	PR	6 m	—
Present study (2024)	Case report	1	Erdafitinib	SD	5.5 m	DOD 15 m after starting Erdafitinib

DCR, disease control rate; DOD, dead of disease; m, months; *N*, number of patients; ORR, objective response rate; OS, overall survival since the start of FGFR inhibitors; PFS, progression-free survival since the start of FGFR inhibitors; PD, progressive disease; pts, patients; SD, stable disease; TMZ, temozolomide.

Despite these promising signs of activity in patients with gliomas, to our knowledge, it has not been properly evaluated whether erdafitinib traverses the blood-brain barrier.^20^ Several smaller studies have demonstrated the activity of erdafitinib and other FGFR inhibitors in patients with FGFR-altered gliomas (Table 1).^4,5,9,11,16,18^ In a small phase II trial of 13 patients, DCR with pemigatinib was 46.2% with a median OS of 6.1 months.^17^ Interestingly, Liu et al. described the case of a patient with and FGFR3-fusion positive GB achieving a partial response during treatment with the FGFR inhibitor pemigatinib.^19^ Nevertheless, objective responses with FGFR inhibitors in gliomas, particularly in HGG, are low. Disease stabilizations are more common and may be durable in some patients. This was shown in the present case, who remained for 5.5 months of on-treatment. While disease stabilization was shorter than 6 months, we demonstrated a clear deceleration in tumor growth during treatment with erdafitinib when compared to the prior and post-erdafitinib treatment periods (Table 1, Figure 2, Supplementary Figure S1). Erdafitinib has also been evaluated in pediatric FGFR-altered CNS tumors, although the FGFR actionability may depend on the entity being treated. For example, in a recent report on two cases of pediatric posterior fossa ependymomas, there were no responses, while a third patient harboring an IDH-WT GB achieved durable disease stabilization.^15,21^

Other pan-FGFR inhibitors are being evaluated in different solid tumors such as futibatinib, derazantinib, and rogaratinib, although no data are yet available on their safety or efficacy in gliomas.^22,23^ Given the growing recognition of FGFR fusions as an important target in IDH-WT GBs, recently the EANO Guideline on targeted treatments recommended to “consider” treatment with FGFR inhibitors in these tumors, although, due to the limited evidence to date, to do so preferably within a clinical trial or within a prospective registry.^24^

Much is still unknown regarding the biology of FGFR-altered gliomas. In our patient, the three tumor biopsies obtained over 4 consecutive years, demonstrate, as expected, an increasing tumor aggressiveness through time with an increase in Ki67 proliferation index and the appearance of cellular pleomorphism and necrosis (Figure 1). Although EGFR alterations have been claimed to be mutually exclusive with FGFR fusions in gliomas, in the biopsy from initial diagnosis NGS revealed an EGFR amplification, that disappeared in the two subsequent biopsies. Whether EGFR and FGFR are mutually exclusive from early steps of the disease or if it is a result of disease evolution is currently unknown.^3,4^ Although we could not study EGFR alterations by means of other techniques such as immunohistochemistry or in situ hybridization, the NGS analysis performed is considered highly sensitive for the detection of any known pathogenic EGFR alteration such as amplifications, mutations, or fusions.^13^ The FGFR3-TACC3 fusion and the TERT promoter mutation were retained through time, but other alterations such as the commonly reported CDK4 amplification disappeared while CDKN2A/B loss occurred in the last two tumor biopsies. Although both CDK4 amplifications and loss of CDKN2A/B have been largely described in gliomas, they do not seem to cooccur. This is possibly due to their redundant impact on the cell cycle functioning of cancer cells and would explain the findings in our study.^25^ Unfortunately, CDK alterations have not been shown to be predictive of response to CDK inhibitors in most solid tumors, and CDK inhibitors tested to date have not shown benefit in clinical studies in patients with gliomas.^25,26^

The TP53 mutation locus changed from E286D to R175H in the last two biopsies. Interestingly, TP53 immunohistochemical staining was nearly absent in the first biopsy but clearly present in the last two. Therefore, the TP53 R175H mutation as well as the CDKN2A/B and MTAP exons 5-8 losses seem to be acquired events through time, the latter two being markers of GB progression and poor prognosis.^16,27,28^ Two patients with glioma whose tumors harbored 2 different FGFR alterations -FGFR3 fusion and FGFR3 mutation- have been described, highlighting the complex biology of these tumors.^29^

Several variants of unknown significance (VUS) were reported in the last two biopsies from 2022 -before erdafitinib- and 2023 -after erdafitinib-. While 4 VUS were retained, 2 other VUS were lost, and 4 other new VUS appeared in the 2023 biopsy (Supplementary Table S1).

To our knowledge evidence is lacking regarding possible resistance mechanisms in FGFR-altered gliomas. Facchinetti et al.,^30^ in patients with bladder cancer treated with FGFR inhibitors, described the occurrence of FGFR mutations in the tyrosine kinase domain as well as co-existing alterations in the PI3K-mTOR -including TSC1 and TSC2 mutations- and EGFR pathways, all likely responsible for primary or acquired resistance to FGFR inhibitors. In our study, a VUS in TSC2 was detected in the post-erdafitinib biopsy (Supplementary Table S1). However, neither the pathogenic alterations nor the VUS detected in neither the pre-erdafitinib nor the post-erdafitinib biopsies have been clearly associated with resistance mechanisms to FGFR inhibitors.

Erdafitinib had to be stopped due to a 6 mm intratumor hemorrhagic focus. While GBs are known to suffer from spontaneous hemorrhagic events due to their highly vascularized and pro-angiogenic nature, it was considered safer to stop erdafitinib since a causal relationship could not be disregarded due to a potential anti-VEGFR2 effect (In in vitro assays, erdafitinib was shown to be a potent inhibitor of FGFR1-4 but a weaker inhibitor of VEGFR2 kinase).^31^

Our study has several limitations. Besides being a single case report, we could not evaluate other molecular markers related to the FGFR pathway in GB such as FGF ligands—heparan sulfate and extracellular matrix molecules—, DOCK1 or SOS/RAS, RAS/RAF/MEK/ERK and FRS2–GRB2–SOS–SHP2 pathways, and neither those targets against which erdafitinib shows some activity in vitro, such as VEGFR2.^31–33^

FGFR3-TACC3 fusion-positive IDH-wild-type GB is a rare entity harboring a moderately better prognosis compared to non-FGFR-altered IDH-WT GB. The current case report demonstrated a durable disease stabilization with a slowdown in tumor growth dynamics during treatment with the FGFR inhibitor erdafitinib

## Supplementary Material

vdae139_suppl_Supplementary_Figure_S1

vdae139_suppl_Supplementary_Tables

vdae139_suppl_Supplementary_Materials

## Data Availability

The datasets used and/or analyzed during the current study are available from the corresponding author upon reasonable request.
