# Supplementary figures and images for: Durable benefit and slowdown in tumor growth dynamics with erdafitinib in a FGFR3-TACC3 fusion-positive IDH-wild type glioblastoma
Source: Neurooncol Adv. 2024 Aug 6;6(1):vdae139. doi: 10.1093/noajnl/vdae139 (PMC11358818; doi:10.1093/noajnl/vdae139)

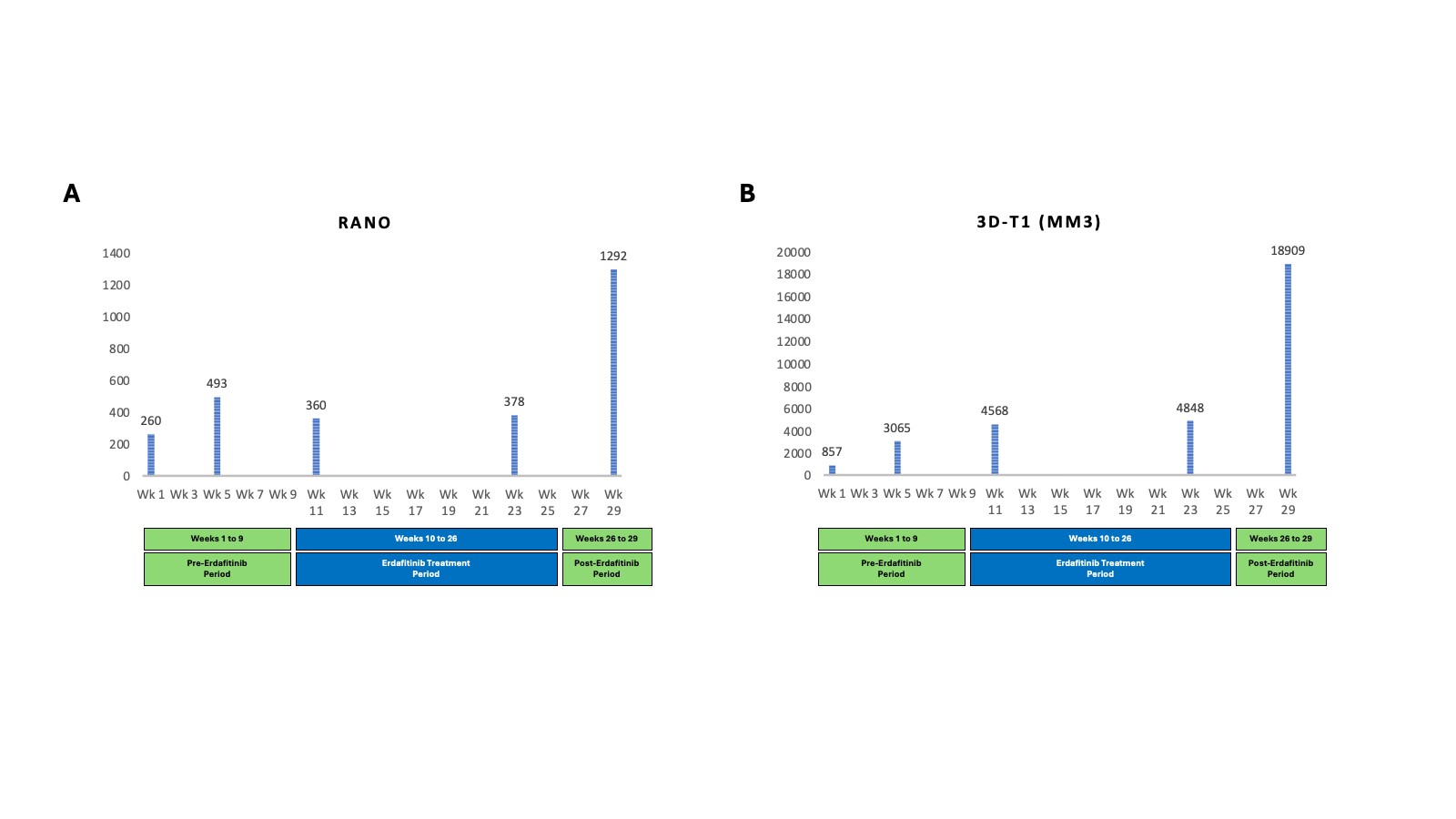

Supplement: vdae139_suppl_Supplementary_Figure_S1 [file vdae139_suppl_supplementary_figure_s1.jpeg]
