# Supplementary material for: Durable benefit and slowdown in tumor growth dynamics with erdafitinib in a FGFR3-TACC3 fusion-positive IDH-wild type glioblastoma
Source: Neurooncol Adv. 2024 Aug 6;6(1):vdae139. doi: 10.1093/noajnl/vdae139 (PMC11358818; doi:10.1093/noajnl/vdae139)
Supplement: vdae139_suppl_Supplementary_Materials [file vdae139_suppl_supplementary_materials.docx]

**SUPPLEMENTARY MATERIAL**

**Table S1.** Variants of unknown significance detected in the 2022 (pre-erdafitinib) and 2023 (post-erdafitinib) tumor biopsies.

**Figure S1.** Tumor dynamics before, during and after treatment with erdafitinib according to RANO criteria and 3D-T1 volumetric measurements.

Temporal evolution of the sum of the products of the perpendicular diameters of target lesions (A) and of tumor volume in mm^3^ (B).

RANO: Response assessment in neuro-oncology, 3D-T1: T1 three-dimensional volume.
